# Trends in CVD Risk Factors for Youth with Incident Diabetes: SEARCH for Diabetes in Youth

**DOI:** 10.1155/2024/5213520

**Published:** 2024-07-11

**Authors:** Ronny A. Bell, Joseph Rigdon, Anna Bellatorre, Dana Dabelea, Ralph D'Agostino, Jasmin Divers, Lawrence M. Dolan, Elizabeth Jensen, Angela D. Liese, Eva Lustigova, Santica M. Marcovina, Lina Merjaneh, David J. Pettitt, Catherine Pihoker, Amy S. Shah, Andrew M. South, Lynne E. Wagenknecht

**Affiliations:** ^1^ University of North Carolina at Chapel Hill, Chapel Hill, NC, USA; ^2^ Wake Forest University School of Medicine, Winston-Salem, NC, USA; ^3^ University of Colorado Anschutz Medical Campus, Aurora, CO, USA; ^4^ New York University Langone Medical Center, New York, NY, USA; ^5^ Cincinnati Children's Hospital Medical Center and The University of Cincinnati, Cincinnati, OH, USA; ^6^ University of South Carolina, Columbia, SC, USA; ^7^ Kaiser Permanente Southern California, Pasadena, CA, USA; ^8^ Medpace Reference Laboratories, Cincinnati, OH, USA; ^9^ University of Washington, Seattle, WA, USA; ^10^Retired, Santa Barbara, CA, USA

## Abstract

**Objectives:**

Cardiovascular disease (CVD) is the leading cause of death and disability among persons with diabetes. Early intervention on cardiovascular risk factors (CRFs) is important in reducing CVD burden. The SEARCH for Diabetes in Youth study assessed CRFs in incident cohorts of youth aged <20 years established from 2002 to 2016. *Research Design and Methods*. Regression models assessed trends over each incident year for lipids (total cholesterol (TC), HDL-c, LDL-c, triglycerides (TG), VLDL-c, and non-HDL-c), kidney function (albumin/creatinine ratio (ACR) ≥30 and ≥300, cystatin C, serum creatinine and estimated glomerular filtration rate (eGFR)), systolic and diastolic blood pressure (BP) *z*-scores, BMI *z*-score, waist circumference (WC), and an inflammatory marker (C-reactive protein (CRP)). Models were stratified by diabetes type (type 1 diabetes (T1D), *N* = 4,600; type 2 diabetes (T2D), *N* = 932) and adjusted for age at diagnosis, sex, race/ethnicity, and diabetes duration. An interaction analysis assessed differential time trends by type.

**Results:**

For youth with T1D, all CRFs significantly improved over time, with the exception of ACR > 300, cystatin C, serum creatinine, eGFR, and CRP. For youth with T2D, TC, LDL-c, and non-HDL-c significantly improved, while eGFR, BMI *z*-score, and CRP significantly worsened. Significant differences in trends over time by type were seen for TC, HDL-c, BMI *z*-score, BP *z*-scores, WC, and CRP.

**Conclusions:**

Overall, improvements in CRFs were more often observed in youth with T1D. Youth with T2D had worsening trends over time in BMI *z*-score, CRP, and kidney function. Further research is needed to better understand these trends and their implications for long-term CVD risk.

## 1. Introduction

Cardiovascular disease (CVD) is the leading cause of death, disability, and health care costs among adults with diabetes [1, 2, 3, 4]. Early detection and management of traditional cardiometabolic risk factors (CRFs), including obesity, blood pressure, lipids, and kidney function, can provide significant benefits in reducing CVD risk, particularly for young adults with diabetes in the early stages of their condition [5].

The SEARCH for Diabetes in Youth study (SEARCH), which has conducted systematic surveillance of incident cases of diabetes in the United States for youth-onset Type 1 diabetes (T1D) and Type 2 diabetes (T2D) (≤19 years of age) since 2002, provides a unique opportunity to monitor trends in health around the time of diagnosis, quality of care and quality of life in this population [6, 7]. The active surveillance period for SEARCH (2002–2020) included monitoring of many events that could potentially influence the quality of care for youth with diabetes, including increased awareness and screening for T2D in youth [8], better treatment options for glycemic control and CRFs [9], and health care reform initiatives, including the Patient Protection and Affordable Care Act, which was signed into law in 2010 but has not been fully implemented nationwide [10].

In a previous SEARCH analysis, Kim et al. [11] examined CRF trends through the 2012 SEARCH incident cohort, showing an increase in the overall number of CRFs among youth with T2D, with an increase in high waist circumference among youth with T2D, and a reduction in the proportion with elevated blood pressure among youth with T1D. The current analysis extends this work by including the most recent SEARCH incident cohort (2016) and an expanded panel of additional CRFs, including measures of kidney function and the inflammatory marker C-reactive protein (CRP) among youth with incident T1D and T2D. We hypothesized that (a) there would be an improvement over time in the absolute levels of CRFs and the proportion with elevated CRFs among youth diagnosed with T1D or T2D, and (b) the rates of improvement would be similar in youth diagnosed with T1D compared to T2D.

## 2. Research Design and Methods

### 2.1. Study Population

Data for this study came from SEARCH, a nationally representative multicenter study of youth with incident T1D or T2D [6, 7]. The SEARCH study included active surveillance in four geographic study sites in South Carolina (the entire state), Ohio (eight counties including Cincinnati), Colorado (the entire state), and Washington (five counties around Seattle), a health plan site (Kaiser Permanente Southern California) and American Indian reservations in Arizona and New Mexico who received their care through the Indian Health Service. Incident cohorts of youth diagnosed at age <20 years (T1D) and age 10–19 years (T2D; ages truncated due to small cell counts for T2D <10 years of age) in these regions were established annually from 2002 to 2006, and subsequently in 2008, 2012, and 2016. Written informed consent was obtained from all participants aged 18 and older. Informed consent as well as parental assent were obtained from the parents or guardians of all participants <18 years of age and participants themselves, respectively, following Institutional Review Board (IRB) regulations at each of the clinical sites. IRB approval was also obtained at the SEARCH Coordinating Center.

### 2.2. Data Collection

Incident cases, ages 3–19 years, identified in the SEARCH catchment areas were invited for an in-person visit to complete questionnaires and for collection of anthropometric data and blood and urine samples by centrally trained research staff under a study-wide protocol to assess clinical outcomes. For youth with incident diabetes in cohorts prior to 2016, all cases were invited for an in-person visit. For participants with incident diabetes in 2016, a subset was invited for the in-person visit. Youth eligible for this visit included all cases diagnosed during 2016 who identified as a racial/ethnic minority, those with a provider diagnosis of T2D, and 25% of non-Hispanic White youth with a provider diagnosis of T1D, randomly selected for invitation.

Blood and urine samples were shipped to the SEARCH Central Laboratory at the Northwest Lipid Metabolism and Diabetes Research Laboratories at the University of Washington, and all data were sent and analyzed by the SEARCH Coordinating Center at the Wake Forest University School of Medicine. Written informed consent was obtained from participants ≥18 years of age and from parents or guardians of younger participants. Written assent was obtained from youth aged 8–17 years. The eight incident cohorts from 2002 to 2006, 2008, 2012, and 2016 generated 11,056 incident cases (8,482 T1D, 2,359 T2D, 205 missing type). Of these, a total of 4,600 individuals with T1D and 932 individuals with T2D completed the in-person visit. Study participants ranged from 438 in 2016 to 699 in 2008 for T1D and from 75 in 2005–209 in 2016 for T2D, with the differences in sample size for 2016 reflective of the change in the recruitment strategy for this cohort.

### 2.3. Study Variables

For the CRFs of interest, this analysis focused on traditional CRFs, including lipid, kidney, inflammation, anthropometric, and blood pressure outcomes. We did not include measures of glycemia as this work has been reported previously in SEARCH for the most recent incident cohort [12, 13]. Lipid outcomes were assessed from blood samples drawn after at least an 8-hr fast and included total cholesterol (TC), HDL cholesterol (HDL-c), LDL cholesterol (LDL-c), triglycerides (TG), very low-density lipoprotein (VLDL) cholesterol, and non-HDL cholesterol. Lipid analyses were performed enzymatically on a Roche c502 autoanalyzer (Roche Diagnostics, Indianapolis, Indiana). LDL-c levels were assessed by the Friedewald equation for study participants with TG levels less than 400 mg/dL [14] and by the Lipid Research Clinics Beta Quantification [15] for those with TG levels of ≥400 mg/dL. Kidney outcomes include serum creatinine, albumin to creatinine ratio (ACR, two separate binary variables as ACR< or ≥30 and ACR ≤ or >300) [16], cystatin C, and estimated glomerular filtration rate (eGFR) as calculated using the 2021 CKD-EPI creatinine-only and creatinine-cystatin C equations [17]. Serum and urinary creatinine were assessed using the Creatinine Plus enzymatic Roche reagent on a Roche c501 Cobas chemistry auto-analyzer (Roche Diagnostics, Inc., Indianapolis, IN). Urinary albumin was assessed from an overnight urine sample using Siemens reagent (Siemens Healthcare Diagnostics, Inc., Newark, DE). Cystatin C was assessed from serum using an immunochemical technique with Siemens reagents (Siemens Healthcare Diagnostics, Inc., Newark, DE). Inflammation was assessed through the measurement of CRP, which was performed using Siemens reagent on a Siemens BNII nephelometer (Siemens Healthineer, Cary, NC).

The anthropometric outcomes of interest were age- and sex-specific BMI *z*-scores, assessed through height and weight measurements collected with participants wearing light indoor clothing without shoes (average of two measures) and waist circumference (WC), measured using the NHANES protocol, with the average of two measures, with a third measure conducted if the initial two measures differed by 1.0 cm [18]. Blood pressure was assessed using a standard sphygmomanometer with size-appropriate cuffs, and measures included systolic blood pressure (SBP) *z*-score and diastolic blood pressure (DBP) *z*-score, both calculated using the 2017 American Academy of Pediatrics Clinical Practice Guideline [19, 20]. Demographic characteristics included age at study visit, age at diabetes diagnosis, and self-reported race/ethnicity (non-Hispanic White, non-Hispanic Black, Hispanic White, and others, including American Indian/Alaska Natives and Asian/Pacific Islanders). For statistical analyses, race/ethnicity was grouped as white, Black, Hispanic, or other. Diabetes type was defined as the type assigned by a health care provider at the time of diagnosis obtained from medical records by SEARCH research staff.

### 2.4. Statistical Analysis

First, we conducted a descriptive analysis of the pooled cross-sectional incident cases, organized by diabetes type. Next, CRFs over time (differences in CRF absolute values and percentages with elevated CRFs across each of the incident groups in 2002–2006, 2008, 2012, and 2016) for various outcomes of interest were displayed graphically by diabetes type. Finally, hypotheses about change over time for outcomes of interest (by type) were formally tested using both unadjusted and adjusted statistical models adjusted for age at diabetes diagnosis, sex, and race/ethnicity. More specifically, we tested for a linear trend for the change in outcomes over time and further examined whether or not this trend differed by diabetes type using an interaction term in the statistical model. Linear regression was conducted for continuous outcomes (TC, HDL-c, LDL-c, TG, VLDL-c, non-HDL cholesterol, serum creatinine, cystatin C, eGFR, BMI *z*-score, SBP *z*-score, and DBP *z*-score) and coefficients are reported for change in outcome for a 1-year increase in time. Logistic regression was conducted for binary outcomes (ACR <30 or ≥30 and ACR ≤300 or >300), and odds ratios are reported for change in odds of outcome for a 1-year increase in time. The statistical significance level was set at *α* = 0.05. Analyses were conducted using R version 4.0.2 [21].

## 3. Results

Table 1 presents data on study participants stratified by diabetes type for the number and percentage of study participants in each incident year, mean (±standard deviation, SD) age at diagnosis and at in-person visit, distribution by sex and race/ethnicity, and mean (±SD) BMI. Compared with participants with T1D, youth with T2D were significantly older at diagnosis and at the study visit, had higher BMI *z*-score, and were more likely to be female and identify with racial/ethnic minority groups (all *p* values <0.0001).

**Figure 1:**
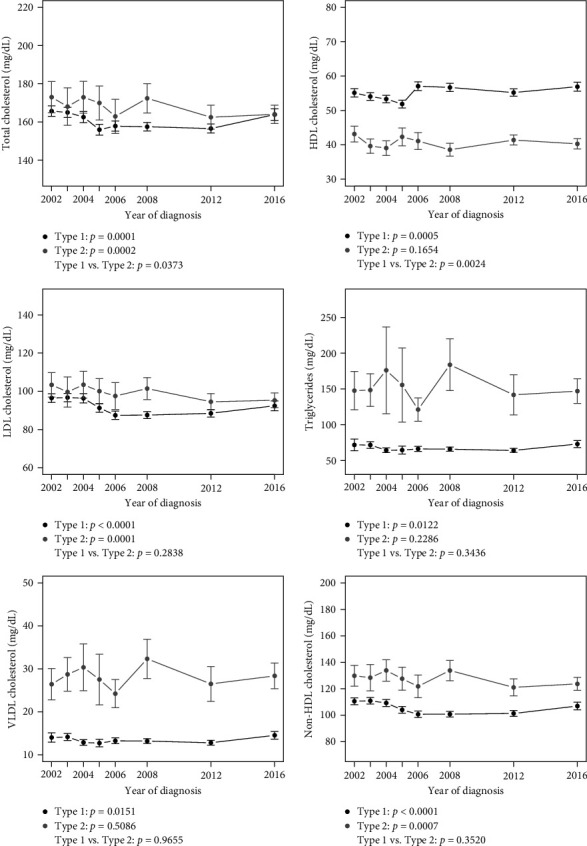
Lipid outcomes over time by diabetes type. Estimates include 95% confidence intervals at each year.

**Figure 2:**
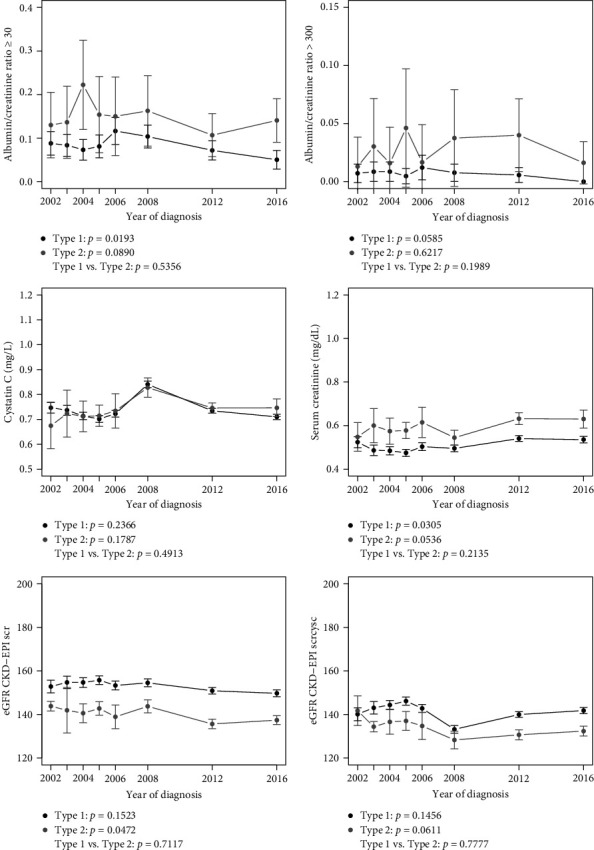
Kidney outcomes over time by diabetes type. Estimates include 95% confidence intervals at each year.

**Figure 3:**
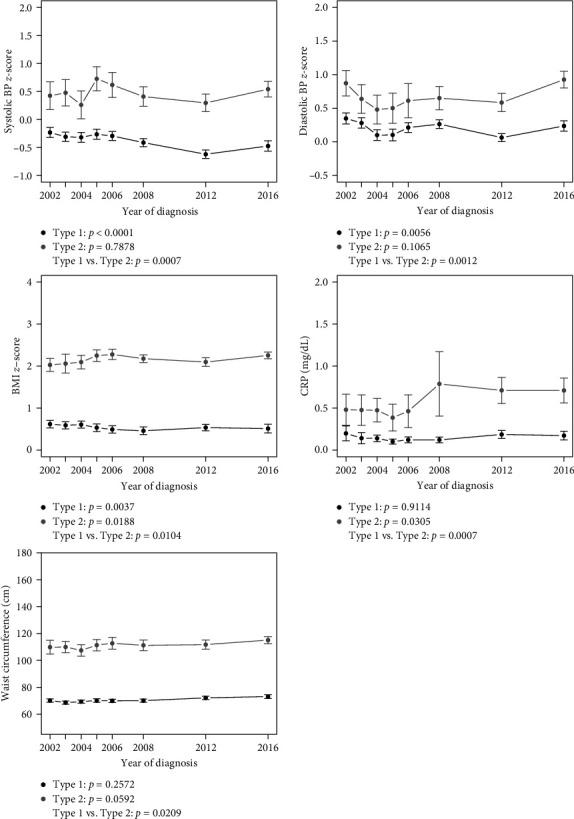
Blood pressure, BMI, C-reactive protein, and waist circumference outcomes over time by diabetes type. Estimates include 95% confidence intervals at each year.

Figures 1, 2, and 3 show linear trends in CRFs, and Table 2 presents point estimates for adjusted models for trends in CRFs for youth by diabetes type and a comparison of trends between types (mean values for each CRF by type and year are provided in Tables S1(a), S1(b), and S1(c)). For youth with T1D, there was a significant trend in improvement for all lipids, SBP, DBP and BMI *z*-scores, and a significant worsening trend for serum creatinine. There was also a significant 3% reduction per year in odds of ACR ≥30 for each increasing year of calendar time. For T2D, there was a significant trend in improvement in TC, LDL-c, and non-HDL-c, and a significant trend in the worsening of eGFR, BMI *z*-score, and CRP.

For the comparison of the change in CRFs over time (across each of the incident groups) between T1D and T2D (Table 2 and Figures 1, 2, and 3), a significant difference was observed for a greater reduction in TC over time T2D vs. T1D (interaction term −0.50, 95% CI: −0.98 to −0.03), and a greater improvement in HDL-c over time in T1D vs. T2D (interaction term 0.29, 95% CI: 0.10–0.48). Conversely, a significant difference between diabetes type in trends over time was observed for SBP *z*-score (interaction term –0.0251, 95% CI: 0.0105–0.0397), DBP *z*-score (interaction term –0.0216, 95% CI: 0.0085–0.0347), BMI *z*-score (interaction term –0.019, 95% CI: 0.0045–0.0336), WC (interaction term –0.2232, 95% CI 0.0339, 0.4125) and CRP (interaction term –0.0182, 95% CI: 0.0077–0.0287), indicating greater reductions over time in blood pressure and BMI *z*-scores in youth with T1D compared to T2D and an increase over time in CRP in T2D compared to T1D.

## 4. Conclusions

CVD exacts a significant toll on individuals with diabetes [1, 2, 3, 4]. The onset of diabetes in the early stages of life increases the likelihood of a cardiovascular event at some point in the lifetime of that individual, making it imperative to assess, monitor, and treat CRFs as soon after diagnosis as possible. This is important given the increasing rates of T2D in adolescents, particularly in racial/ethnic minority populations [22, 23].

In a previous analysis of SEARCH data, Kim et al. [11] examined trends in CRF among youth by diabetes type, including data from 2002 to 2012. They observed that there was a higher prevalence of CRF “clusters” (≥2 CRFs) in youth with T2D compared to youth with T1D, although the prevalence of CRF clusters did not change over time. They also observed that the proportion of high blood pressure decreased over time for youth with T1D, while the prevalence of high waist circumference increased over time for youth with T2D. In this work, we chose to predominantly study quantitative rather than binary outcomes in order to maximize power and use all available data.

This analysis extends the previous SEARCH analysis to include a more recent and more diverse cohort (2016) primarily focused on the analysis of continuous CRF data (with the exception of ACR) and to include other CRFs such as additional lipid measures, an inflammation marker (CRP) and kidney function. We found that there were improvements in most of the CRFs over this time period, more so for youth with T1D, in support of our first hypothesis. There is, however, some level of concern that these risk factors are at a level to put youth at elevated risk for future CVD. For example, for youth with T2D, levels of HDL-c and non-HDL-c were consistently within the range considered unhealthy (≤45 and ≥120 mg/dl, respectively) [24].

With regard to our second hypothesis, our findings were somewhat mixed. The trend in improvement in TC was significantly greater for youth with T2D compared to youth with T1D. Conversely, improvements in HDL-c, blood pressure *z*-scores, and BMI *z*-score were greater in youth with T1D compared to youth with T2D, while CRP worsened over time in T2D vs. T1D. For BMI *z*-score, WC, and CRP, this difference was partially a reflection of an increase in these CRFs in youth with T2D.

These findings offer some areas of optimism and some areas of concern regarding trends associated with CVD risk for youth at the onset of their condition. Youth with T1D demonstrated an improvement in almost all CRFs over time. While kidney function did not change appreciably over time in youth with T1D, the slightly decreased odds of albuminuria are encouraging. While we did observe a statistically significant increase in serum creatinine over time in this population, this is not likely to be clinically significant for three reasons: The estimate is exceedingly small (0.001 mg/dL/year), there was no concomitant statistically or clinically significant increase in cystatin C (a more accurate measure of kidney function compared to creatinine), and our two eGFR measures did not change significantly over time.

Conversely, we observed a troubling pattern of decreased eGFR and increased BMI *z*-score and CRP for youth with T2D. While height and weight are generally measured in clinical encounters, CRP is generally not part of a routine blood panel, so its interpretation here may not be as clinically useful as other CRFs. CRP is also nonspecific and does not guide clinical management. Finally, it is concerning that blood pressure did not decrease over time in youth with T2D, suggesting a need for primary prevention strategies and aggressive treatments in this population to decrease the risk of future CVD.

In light of these findings, this study has a number of limitations that must be taken into consideration. First, this study focused on the surveillance of CRFs in incident diabetes cases rather than a longitudinal assessment of individual cases. Second, this analysis was limited by the CRF variables collected in the SEARCH study at the single study visit soon after diagnosis. Finally, this study relied on the use of diabetes type based on classification in the study participant's medical record or physician reports. However, this does not appear to be a significant concern, as previous analyses of SEARCH data indicate relatively high agreement between provider-assigned diabetes type and etiologically-based diabetes type (94.4%–96.0% for T1D and 73.2%–84.8% for T2D) [22].

However, the study has a number of strengths, including the large sample size of youth with T1D and T2D, their racial/ethnic diversity, a standardized protocol for assessment of clinical variables, and the availability of data over an extended period of time (15 years), which may be informed by increased awareness of T2D in youth, changes in health care legislation and updated treatment guidelines for CRFs in these populations [5, 10].

This study provides unique insights into our understanding of trends in CRFs among youth with T1D and T2D. These trends are occurring within the contexts of an increased awareness of T2D in youth, changes in health care policies impacting access to care, such as the Affordable Care Act, the increased availability of treatment options CVD risk factors for youth with diabetes and both positive and negative trends in lifestyle behaviors related to increased CVD risk. While this study did not examine these relationships, it nonetheless provides important clinical considerations for providers who care for youth with diabetes. Further research is needed to better understand the relationship between youth-onset diabetes and CVD risk to enhance prevention and treatment options to reduce long-term cardiovascular risk.

## Figures and Tables

**Table 1:** Incident year distribution and demographic characteristics of the study sample by diabetes type.

Characteristic	Type 1	Type 2	*p*-Value^1^
	*n* = 4,600	*n* = 932	
Year			
2002	545 (11.8%)	96 (10.3%)	—
2003	601 (13.1%)	86 (9.2%)	—
2004	574 (12.5%)	82 (8.8%)	—
2005	542 (11.8%)	75 (8.0%)	—
2006	562 (12.2%)	76 (8.2%)	—
2008	699 (15.2%)	117 (12.6%)	—
2012	639 (13.9%)	191 (20.5%)	—
2016	438 (9.5%)	209 (22.4%)	—
Age at diagnosis	9.8 (±4.3)	14.5 (±2.6)	<0.0001
Age at in-person visit	10.7 (±4.4)	15.6 (±2.8)	<0.0001
Sex			
Female	2,178 (47.3%)	570 (61.2%)	<0.0001
Male	2,422 (52.7%)	362 (38.8%)	—
Race/ethnicity			
Hispanic	680 (14.8%)	265 (28.4%)	<0.0001
Non-Hispanic Black	575 (12.5%)	400 (42.9%)	—
Non-Hispanic White	3,200 (69.6%)	166 (17.8%)	—
Other	145 (3.2%)	101 (10.8%)	—
BMI (kg/m^2^)	20.0 (±5.1)	35.9 (±8.8)	<0.0001
BMI *z*-score	0.5 (±1.0)	2.2 (±0.7)	<0.0001

*N* (%) or mean (±SD). ^1^From Wilcoxon rank-sum test or Fisher's exact test.

**Table 2:** Tests of trend over time for CRF outcomes by diabetes type and differences in trend by type.

CRF outcome	Type 1 diabetes trend over time		Type 2 diabetes trend over time		Difference in type 2 vs. type 1 diabetes trend over time	
	Estimate (95% CI)	*p*-Value	Estimate (95% CI)	*p*-Value	Estimate (95% CI)	*p*-Value
Lipids						
Total cholesterol (mg/dL)	−0.43 (−0.65, −0.21)	0.0001	−1.02 (−1.56, −0.48)	0.0002	−0.50 (−0.98, −0.03)	0.0373
HDL cholesterol (mg/dL)	0.17 (0.08, 0.27)	0.0005	−0.10 (−0.24, 0.04)	0.1654	−0.29 (−0.48, −0.1)	0.0024
LDL cholesterol (mg/dL)	−0.53 (−0.71, −0.34)	<.0001	−0.82 (−1.23, −0.41)	0.0001	−0.21 (−0.6, 0.18)	0.2838
Triglycerides (mg/dL)	−0.48 (−0.86, −0.1)	0.0122	−1.41 (−3.71, 0.89)	0.2286	−0.61 (−1.89, 0.66)	0.3436
VLDL cholesterol (mg/dL)	−0.08 (−0.14, −0.01)	0.0151	−0.10 (−0.41, 0.20)	0.5086	0.00 (−0.19, 0.18)	0.9655
Non-HDL cholesterol (mg/dL)	−0.60 (−0.81, −0.40)	<.0001	−0.92 (−1.46, −0.39)	0.0007	−0.22 (−0.67, 0.24)	0.3520
Kidney outcomes						
Albumin/creatinine ratio ≥30 mg/mmol ^*∗*^	0.97 (0.94, 0.99)	0.0193	0.96 (0.92, 1.01)	0.0890	1.02 (0.97, 1.07)	0.5356
Albumin/creatinine ratio >300 mg/mmol ^*∗*^	0.90 (0.81, 1.00)	0.0585	0.98 (0.89, 1.07)	0.6217	1.09 (0.95, 1.25)	0.1989
Cystatin C (mg/L)	0.0007 (−0.0005, 0.0019)	0.2366	0.0028 (−0.0013, 0.0068)	0.1787	0.0010 (−0.0019, 0.0040)	0.4913
Serum creatinine (mg/dL)	0.0010 (0.0001, 0.0019)	0.0305	0.0043 (−0.0001, 0.0086)	0.0536	0.0017 (−0.001, 0.0044)	0.2135
eGFR CKD-EPI scr ^*∗∗*^	−0.06 (−0.14, 0.02)	0.1523	−0.25 (−0.51, 0.00)	0.0472	−0.04 (−0.24, 0.16)	0.7117
eGFR CKD-EPI scrcysc ^*∗∗*^	−0.09 (−0.22, 0.03)	0.1456	−0.31 (−0.63, 0.01)	0.0611	−0.04 (−0.33, 0.25)	0.7777
Other CRFs						
SBP *z*-score	−0.0278 (−0.0349, −0.0207)	<.0001	−0.0020 (−0.0162, 0.0123)	0.7878	0.0251 (0.0105, 0.0397)	0.0007
DBP *z*-score	−0.0090 (−0.0154, −0.0026)	0.0056	0.0108 (−0.0023, 0.0239)	0.1065	0.0216 (0.0085, 0.0347)	0.0012
BMI *z*-score	−0.0112 (−0.0187, −0.0036)	0.0037	0.0108 (0.0018, 0.0199)	0.0188	0.019 (0.0045, 0.0336)	0.0104
CRP (mg/dL)	−0.0002 (−0.0046, 0.0041)	0.9114	0.0156 (0.0015, 0.0298)	0.0305	0.0182 (0.0077, 0.0287)	0.0007
Waist circumference (cm)	−0.0442 (−0.1206, 0.0323)	0.2572	0.2689 (−0.0105, 0.5482)	0.0592	0.2232 (0.0339, 0.4125)	0.0209

^*∗*^Odds ratios reported.  ^*∗∗*^See [16].

## Data Availability

Data and data dictionaries, forms, and manuals of procedures for the SEARCH for Diabetes in Youth study are available on the National Institute of Diabetes and Digestive and Kidney Diseases (NIDDK) website at https://repository.niddk.nih.gov/studies/search/.

## Additional Points

*Summary*. Early CVD risk factor (CRF) control is important in reducing CVD burden. The SEARCH study examined CRF trends in youth with incident T1D/T2D from 2002 to 2016. Youth with T1D had better patterns over time vs. youth with T2D. Further research is needed to understand these trends and their implications for long-term CVD risk. *Central Laboratory*. (*PI*) Santica M. Marcovina, PhD, ScD. Alan Chait, MD; Noemie Clouet-Foraison, PhD; Jessica Harting; Greg Strylewicz, PhD for the University of Washington Northwest Lipid Metabolism and Diabetes Research Laboratories. *Coordinating Center*. (Co-PIs) Ralph D'Agostino, Jr., PhD, Elizabeth T. Jensen, MPH, PhD; Lynne E. Wagenknecht, DrPH; Ramon Casanova, PhD; Jasmin Divers, PhD; Maureen T. Goldstein, BA; Leora Henkin, MPH, M.Ed; Scott Isom, MS; Kristin Lenoir, MPH; June Pierce, AB; Beth Reboussin, PhD; Joseph Rigdon, PhD; Andrew M. South, MD, MS; Jeanette Stafford, MS; Cynthia Suerken, MS; Brian Wells, MD, PhD; Carrie Williams, MA, CCRP for Wake Forest University School of Medicine.

## Supplementary Materials

Supplementary Materials Table S1(a–c): CRF outcomes over time by diabetes type.
