# Supplementary material for: Trends in CVD Risk Factors for Youth with Incident Diabetes: SEARCH for Diabetes in Youth
Source: Pediatr Diabetes. 2024 Jul 11;2024:5213520. doi: 10.1155/2024/5213520 (PMC12017249; doi:10.1155/2024/5213520)
Supplement: Supplementary Materials — Table S1(a–c): CRF outcomes over time by diabetes type. [file 5213520.f1.docx]

Supplement Table 1a**:**  Lipid outcomes over time by diabetes type

|  | Type | 2002 | 2003 | 2004 | 2005 | 2006 | 2008 | 2012 | 2016 |
| --- | --- | --- | --- | --- | --- | --- | --- | --- | --- |
| Total cholesterol (mg/dL) | 1 | 165.7 | 165.0 | 162.6 | 155.9 | 157.8 | 157.5 | 156.5 | 163.9 |
|  | 2 | 173.0 | 168.0 | 172.9 | 169.9 | 162.9 | 172.4 | 162.5 | 164.0 |
| HDL cholesterol (mg/dL) | 1 | 55.1 | 54.1 | 53.3 | 51.9 | 57.1 | 56.7 | 55.2 | 56.9 |
|  | 2 | 43.1 | 39.6 | 39.2 | 42.3 | 41.1 | 38.6 | 41.4 | 40.3 |
| LDL cholesterol (mg/dL) | 1 | 96.5 | 96.8 | 96.4 | 91.3 | 87.5 | 87.6 | 88.5 | 92.4 |
|  | 2 | 103.4 | 99.7 | 103.5 | 100.1 | 97.6 | 101.5 | 94.6 | 95.3 |
| Triglycerides (mg/dL) | 1 | 71.8 | 71.7 | 64.4 | 64.7 | 66.3 | 65.9 | 64.1 | 73.1 |
|  | 2 | 147.7 | 148.5 | 176.0 | 155.6 | 121.2 | 183.9 | 141.8 | 147.0 |
| VLDL cholesterol (mg/dL) | 1 | 14.0 | 14.2 | 12.9 | 12.7 | 13.3 | 13.2 | 12.8 | 14.6 |
|  | 2 | 26.4 | 28.7 | 30.4 | 27.5 | 24.3 | 32.4 | 26.5 | 28.4 |
| Non-HDL cholesterol (mg/dL) | 1 | 110.5 | 110.9 | 109.2 | 104.1 | 100.7 | 100.8 | 101.3 | 106.9 |
|  | 2 | 129.8 | 128.4 | 133.9 | 127.6 | 121.8 | 133.8 | 121.1 | 123.7 |

Supplement Table 1b**:**  Kidney outcomes over time by diabetes type

|  | Type | 2002 | 2003 | 2004 | 2005 | 2006 | 2008 | 2012 | 2016 |
| --- | --- | --- | --- | --- | --- | --- | --- | --- | --- |
| Albumin/creatinine ratio >30 mg/mmol | 1 | 0.09 | 0.08 | 0.07 | 0.08 | 0.12 | 0.10 | 0.07 | 0.05 |
|  | 2 | 0.13 | 0.14 | 0.22 | 0.15 | 0.15 | 0.16 | 0.11 | 0.14 |
| Albumin/creatinine ratio >300 mg/mmol | 1 | 0.01 | 0.01 | 0.01 | 0.00 | 0.01 | 0.01 | 0.01 | 0.00 |
|  | 2 | 0.01 | 0.03 | 0.02 | 0.05 | 0.02 | 0.04 | 0.04 | 0.02 |
| Cystatin C (mg/L) | 1 | 0.75 | 0.74 | 0.71 | 0.70 | 0.72 | 0.84 | 0.73 | 0.71 |
|  | 2 | 0.67 | 0.72 | 0.71 | 0.71 | 0.73 | 0.83 | 0.75 | 0.75 |
| Serum creatinine (mg/dL) | 1 | 0.52 | 0.49 | 0.48 | 0.47 | 0.50 | 0.50 | 0.54 | 0.54 |
|  | 2 | 0.55 | 0.60 | 0.57 | 0.58 | 0.61 | 0.54 | 0.63 | 0.63 |
| eGFR CKD-EPI scr | 1 | 152.9 | 154.7 | 154.7 | 155.8 | 153.3 | 154.5 | 150.9 | 149.7 |
|  | 2 | 143.8 | 141.9 | 140.6 | 142.8 | 138.9 | 143.7 | 135.6 | 137.4 |
| eGFR CKD-EPI scrcysc | 1 | 140.1 | 143.1 | 144.4 | 146.2 | 142.8 | 133.2 | 140.0 | 141.8 |
|  | 2 | 141.7 | 134.4 | 136.6 | 137.0 | 134.7 | 128.3 | 130.6 | 132.4 |

Supplement Table 1c**:**  Blood pressure, BMI, C-reactive protein, and waist circumference outcomes over time by diabetes type

|  | Type | 2002 | 2003 | 2004 | 2005 | 2006 | 2008 | 2012 | 2016 |
| --- | --- | --- | --- | --- | --- | --- | --- | --- | --- |
| SBP z-score | 1 | -0.23 | -0.31 | -0.32 | -0.26 | -0.29 | -0.41 | -0.62 | -0.47 |
|  | 2 | 0.42 | 0.48 | 0.26 | 0.73 | 0.62 | 0.41 | 0.30 | 0.54 |
| DBP z-score | 1 | 0.35 | 0.28 | 0.10 | 0.10 | 0.21 | 0.26 | 0.06 | 0.24 |
|  | 2 | 0.87 | 0.64 | 0.48 | 0.50 | 0.61 | 0.65 | 0.59 | 0.93 |
| BMI z-score | 1 | 0.62 | 0.59 | 0.61 | 0.53 | 0.49 | 0.46 | 0.53 | 0.51 |
|  | 2 | 2.03 | 2.06 | 2.09 | 2.25 | 2.28 | 2.18 | 2.10 | 2.25 |
| CRP (mg/dL) | 1 | 0.20 | 0.14 | 0.14 | 0.10 | 0.12 | 0.12 | 0.19 | 0.17 |
|  | 2 | 0.48 | 0.48 | 0.47 | 0.39 | 0.46 | 0.79 | 0.71 | 0.71 |
| Waist circumference (cm) | 1 | 70.18 | 68.66 | 69.34 | 70.16 | 69.92 | 70.10 | 72.14 | 73.11 |
|  | 2 | 109.9 | 110.0 | 107.5 | 111.4 | 112.8 | 111.2 | 111.8 | 115.1 |
